# From Discourse to Practice—Facilitating Factors and Barriers to the Implementation of Pharmaceutical Care in Primary Health Care: A Qualitative Study

**DOI:** 10.3390/pharmacy14030067

**Published:** 2026-04-30

**Authors:** Jéssica Azevedo Aquino, Denise Alves Guimarães, Mariana Linhares Pereira, Luanna G. Resende Silva, João Pedro Vasconcelos Paolinelli, André Oliveira Baldoni

**Affiliations:** 1 Department of Occupational Health Care, Federal University of Minas Gerais (UFMG), Belo Horizonte 31270-901, MG, Brazil; 2 Postgraduate Program in Health Sciences, Federal University of São João del-Rei (UFSJ), Divinópolis 355001-296, MG, Brazilandrebaldoni@ufsj.edu.br (A.O.B.); 3 Faculty of Medicine, Federal University of São João del-Rei (UFSJ), Divinópolis 355001-296, MG, Brazil

**Keywords:** pharmaceutical services, primary health care, pharmacists, qualitative research, implementation science, education continuing

## Abstract

Background: The implementation of pharmaceutical care (PC) in Primary Health Care (PHC) faces challenges related to interprofessional integration, management support, and infrastructure, despite its potential to improve patient-centered care. Objectives: To analyze, from the perspective of pharmacists working in PHC, the facilitating factors and barriers to the implementation of PC in the public health system in a post-implementation context. Methods: This is a qualitative, exploratory study, with eight individual semi-structured interviews conducted remotely between December 2024 and February 2025. The interviews were recorded, transcribed, and subjected to thematic content analysis by a trained team, with peer validation. Results: The main facilitators identified were: a solid relationship with the team, participation in continuing education, and a bond with patients. Barriers included a lack of adequate infrastructure, workload, staff turnover, and resistance from the health team. Management support, although frequently cited as a facilitator, did not always translate into concrete actions for service implementation. Even in the face of these barriers, the implementation of PC contributed to strengthening patient-centered care, the clinical protagonism of pharmacists, and improving health outcomes. Conclusions: The analysis highlighted that the sustainability of PC depends on structural and relational conditions that go beyond individual training. The study reveals that, after implementation and continuing education processes, organizational barriers persist that limit the consolidation of PC, highlighting the need for institutional policies and ongoing management support to effectively integrate pharmacists into healthcare.

## 1. Introduction

Pharmaceutical care (PC) directly impacts the reduction in health harm and costs for public systems [1,2,3,4]. This practice is essential in the Brazilian context, where the public health system ensures universal, comprehensive, and free access to health services and medications for the population [5]. In Brazil, PC has been progressively recognized as a component of person-centered care, with the pharmacist integrated into the healthcare team and co-responsible for the therapeutic process [6,7].

In Brazil, Primary Health Care (PHC), the main gateway to the health system, is organized primarily through the Family Health Strategy (FHS), which operates in a territorialized and multidisciplinary manner [8]. Recently, the composition of these teams has expanded with the creation of Multidisciplinary Teams, which include pharmacists, psychologists, nutritionists, physiotherapists, and others, aiming to broaden the scope and resolution of the service [9]. In this context, the role of pharmacists has evolved from functions focused on medication logistics to an increasingly clinical role integrated into healthcare teams [10,11]. This clinical role includes activities such as pharmaceutical consultation, the identification and management of medication-related problems, health education, pharmacotherapeutic follow-up, and collaboration with other professionals in shared care [6,7].

PC has demonstrated positive impacts on treatment adherence, user satisfaction, and clinical outcomes, especially among the elderly, and people with diabetes mellitus, systemic arterial hypertension, cardiovascular disease, dyslipidemia, asthma, depression, and heart failure [12,13,14,15]. However, many of these experiences occur under the supervision of research or technical support teams, and when this support ends, it leads to the discontinuation of clinical follow-up and worsening of patients’ health parameters [16,17,18].

This fragility highlights the urgent need for strategies that consolidate the implementation of PC on a permanent and lasting basis, leveraging professionals already working in municipal health networks. Studies indicate that a significant portion of Brazilian community pharmacists may not be sufficiently prepared to practice clinically [19,20], highlighting the need for Continuing Professional Development (CPD) linked to daily practice [21].

Several initiatives have sought to respond to this challenge, such as the ImplanFarSUS project, which aims to support the implementation of PC in PHC through theoretical and practical training and technical supervision [22,23]. However, studies analyzing what remains after the end of these processes are still scarce, especially from the perspective of the pharmacists themselves, who are integrated into these services.

Therefore, this study aims to analyze, from the perspective of pharmacists working in PHC, the facilitating and hindering factors for implementing PC in the healthcare system, following a CPD process and implementation of PC.

## 2. Methods

### 2.1. Study Design

A qualitative, exploratory study was conducted based on interviews with pharmacists who underwent the process of implementing PC within the healthcare system—ImplanFarSUS in different municipalities in the state of Minas Gerais, Brazil [22]. ImplanFarSUS consisted of a training and implementation-support initiative for pharmaceutical care in PHC, involving theoretical-practical training for pharmacists, followed by the implementation of pharmacotherapeutic follow-up through pharmaceutical consultations, with monitoring and technical support from the researchers throughout the process [23].

As the final stage of the project, all participating pharmacists were invited to take part in the qualitative phase of the investigation, regardless of whether they had completed the planned care activities. No financial incentive was provided for participation in the project. The study was written following the recommendations of the “Consolidated Criteria for Reporting Qualitative Research (COREQ): a 32-item checklist for interviews and focus groups” [24]. The inclusion criteria for the qualitative study were: being a pharmacist participating in ImplanFarSUS (regardless of whether or not they had completed the PHC user care provided by ImplanFarSUS), and also working in the municipal pharmacy.

### 2.2. Development of the Interview Script

**Figure 1:**
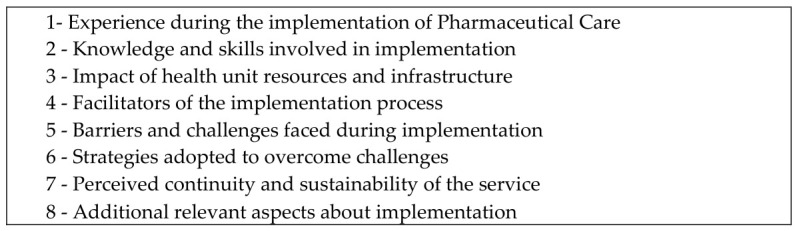
Guiding questions used to structure the semi-structured interviews with pharmacists participating in ImplanFarSUS.

To conduct the interviews, the researchers developed a semi-structured script [25]. The questions in the script were formulated by JAA (female, PhD) and AOB (male, PhD) and refined by DAG and MLP (both female, PhD researchers with prior experience in qualitative study design). JAA, who conducted the interviews and had no prior relationship with the participants, received training from an experienced researcher (DAG) to conduct the interviews. The interview script included questions related to: (1) experience implementing PC; (2) impact of knowledge and skills; (3) resources and infrastructure of the health unit; (4) strengths and facilitators of PC implementation; (5) hindering aspects and/or challenges faced during the implementation process; (6) strategies adopted to overcome challenges; and (7) continuity or lack thereof of PC in their municipality. The guiding questions that informed the interview script are presented in Figure 1.

### 2.3. Interviews

Participants were invited by email, and up to three contact attempts were made via a messaging app. Participants were presented with the study objectives, the researcher’s reasons for conducting the research, and ethical considerations. Interviews were scheduled and conducted individually and remotely between December 2024 and February 2025 via the Google Meet^®^ platform, which was also used for audio and video recording [26]. Of the 17 eligible pharmacists identified and invited to participate in the study, two declined to participate, and seven did not respond to contact attempts. Among the pharmacists who did not respond to contact attempts, none had completed the planned activities of the ImplanFarSUS project.

To ensure the anonymity of the interviewees, an alphanumeric coding system was used, beginning with the initials “F” followed by a number that defines the interviewee, which can range from one to eight (e.g., F1). Information that could jeopardize the anonymity of the interviewees was removed.

### 2.4. Data Analysis

The audio and video content was transcribed by one of the researchers (JAA) using the Fireflies.ia^®^ tool. The transcripts were fully reviewed to ensure fidelity to the original content. Field diaries, audio, and videos were reviewed to capture potential phenomena not highlighted in the transcripts [27]. Microsoft Word^®^ was used for data management.

The material was initially coded by one of the researchers (JAA), with the support of two other researchers (DLG and MLP). One of the researchers (DAG), with prior experience of applying the technique, actively contributed by discussing the analysis throughout the process.

The analysis of the interviews was conducted based on the Content Analysis framework, with a focus on thematic or categorical analysis, as proposed by Bardin (2016) [28]. The process followed three main stages: (1) transcription, re-reading, and organization of the reports; (2) classification of the data by the structure of relevance of the central ideas and the development of categories; and (3) final analysis, establishing connections between the data, the study objective, and the guiding interview questions, in order to deepen the discussion of the findings. From this analysis, two main categories emerged: (1) Challenges for the implementation of PC; (2) potentialities and impacts of structuring PC.

### 2.5. Ethical Aspects

The study was approved by the Research Ethics Committee Involving Human Subjects (CEPES) of the Federal University of São João del-Rei (UFSJ), Dona Lindu Central-West Campus, under opinion CAAE 45666921.0.0000.5545.

## 3. Results

Eight pharmacists from different municipalities participated in the study. The interviews lasted an average of 60 min. The demographic and educational characteristics of the participants are presented in Table 1. The most frequent salary range was 2 to 3 minimum wages, reported by 4 participants (50.0%). Of the eight participants, only one had not completed all the proposed ImplanFarSUS services.

**Figure 2:**
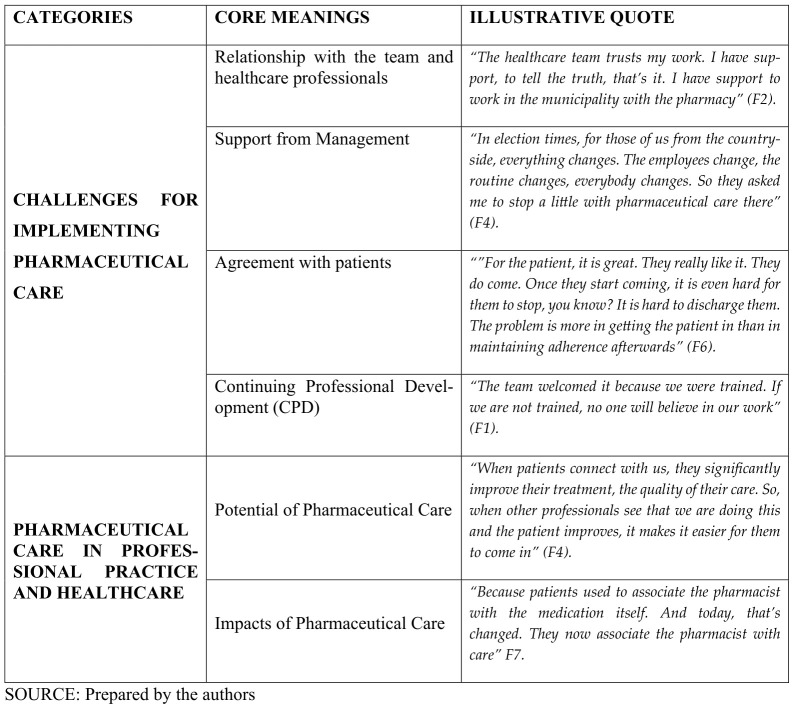
Categories and their respective core meanings.

Figure 2 presents the categories organized by similarity of content and the core meanings that comprise them, with one illustrative participant quote for each nucleus of meaning.

### 3.1. Challenges for Implementing Pharmaceutical Care

The interviewees’ perceptions reveal a set of aspects that make up a complex reality and must be considered to advance the structuring of healthcare. In this sense, the challenges for implementing PC involve the relationship between the pharmacist and other team professionals; infrastructure issues; support from managers; patient agreements; and issues related to CPD.

Regarding the relationship between pharmacists and healthcare team professionals, it was observed that the PC proposal was better received by teams where the pharmacists had **more experience** in the municipality and reported a good relationship with the **team and professionals**.

*“The healthcare team trusts my work. I have support, to tell the truth, that’s it. I have support to work in the municipality with the pharmacy. Support as a professional. The issue of management support, this assistance, this support from the multidisciplinary team, they believe in the service, they have this trust in referring patients to me, the patients do too. Because the population has this access to me, as a pharmacist. So, that helps a lot too. This patient trust. The patient needs to have this trust in the professional, this credibility.”*

(F2)

In contrast, professionals with **less time on the team** and with interprofessional relationships that needed strengthening faced greater challenges in accepting the proposal.

*“And I also emphasize that if there isn’t good communication with the team, the service doesn’t work. It doesn’t work because they don’t know you’re there, they don’t know what your service is. So, if you don’t have them present reinforcing, showing what you do, showing what you did it for, you will become invisible.”*

(F1)

**The realities of the municipalities** and the teams in which the pharmacists were involved also varied greatly. Some municipalities had the multidisciplinary team system in PHC (eMulti), some with already institutionalized PC, and others not. Participants also described differences in physicians’ availability and professional conduct, which, according to their reports, affected the implementation process.

*“[I] work in two units, the doctor has a pre-set schedule, so while he was seeing patients, I wasn’t there. Often, to meet with the doctor, I had to wait until he was off duty to talk to him about the patient. There was a resolution, the doctor saw him, but I wanted feedback, you know? I didn’t get that feedback. I had to go to the doctor to find out.”*

(F4)

**Regarding the relationship of the other professionals on the multidisciplinary healthcare team** regarding the implementation of the PC service, the interviewees reported that some professionals were supportive, while others were more resistant. Furthermore, they reported challenges in getting other healthcare professionals on the teams to refer patients to PC.

*“The nurses were the ones who referred patients… The nutritionist never referred patients, nor did the doctor. So, the doctor supported me by running the tests, right? Sometimes, I asked him about some things, and seeing patients to adjust their doses. But then, I think it’s a lack of understanding of the importance of the service, right? By the professional, by the nutritionist, for example. Because I’m not going to say that the psychologist, the other professionals, sometimes they don’t know much about it, right? They don’t really understand the severity of diabetes. But the nutritionist understands… And they need a specific workload for these appointments, right?”*

(F5)

The interviewees’ reports reveal that **management support**, while frequently cited as a driver of PC, didn’t always translate into concrete, material conditions for the service’s implementation. The analysis of the interviews revealed contradictions regarding what actually constitutes this support, which manifested itself in various forms—from structural actions such as specific hiring of clinical pharmacists, defined workloads for the activities provided by PC, and adequate infrastructure, to generic discourses encouraging management to implement PC, without, however, corresponding to any real changes in working conditions that would enable or encourage its implementation. This support (or lack thereof) spans multiple spheres—federal (with policies and guidelines), municipal, and the local management of the units themselves—and directly impacts the possibility of consolidating PC in PHC.

*“Regarding management, I had a lot of support. They have great confidence in my work. […] But when I received the notice that I could no longer perform capillary blood glucose testing due to the unsafe conditions, I was disappointed.”*

(F2)

*“Today, for example, we have an opening for a pharmacist, right? But they’re hired through a selection process without any criteria. So, a pharmacist arrives who’s never seen a patient before, doesn’t know what clinical pharmacy is. Unfortunately, they end up staying at the unit doing nothing. This leads to questions like, ‘What is the pharmacist doing here? What is the pharmacist’s job? Why is the pharmacist at the unit?’”*

(F6)

Regarding infrastructure, for example, with the exception of one interviewee, in all other municipal settings, it was clear that **management support did not translate into adequate structural conditions** for carrying out the consultations planned in the PC project. The situations reported ranged from the need to adapt makeshift environments for clinical care to the (sometimes conflicting) rotation of rooms with other healthcare professionals.

*“There have even been some suggestions for us to provide services in other locations, but I think it disrupts the relationship, and these are usually locations that aren’t under the jurisdiction of the city, so how can I bring a computer there that has access to patient records? So, providing services outside the FHS (Family Health Strategy), I think, completely undermines the multidisciplinary team’s services; it becomes a specialized service, and that’s not our goal.”*

(F7)

Regarding workload, even with “management support”, it was observed that, among the eight participating pharmacists, only two were dedicated exclusively to clinical activities. Most had to reorganize their routines and adapt to existing demands to facilitate PC services. It’s worth noting that the **workload issues** reported by some participants for performing PC-related activities created overload and hindered the performance of such activities:

*“I had to organize my work schedule, my routine. Because when it was time to see patients, I had to stop doing some things. I had to take work home a few times.”*

(F8)

**Managerial turnover and political and administrative instability** also emerged as factors that directly impacted the continuity of PC. Changes in Mayors or health secretaries, especially during election periods, frequently led to service interruptions or uncertainty about their continued existence.

*“With the change of Mayor, I don’t know how it will continue. Because you don’t know who will be the health secretary.”*

(F4)

Regarding **patient agreement**, pharmacists’ reports suggested that adherence varied among the individuals followed in the service. According to the interviewees, although some patients showed interest and developed a bond with the service over the follow-up period, others appeared to have difficulty understanding the pharmacist’s role, showed limited perception of the seriousness of their health condition, or had restricted availability to attend appointments. From the pharmacists’ perspective, adherence, when achieved, tended to be positive and sustained, but required continuous efforts to raise awareness, build rapport, and adapt communication and care strategies to each patient’s particularities.

*“At first, I don’t think they really understood where this was going, you know? … I had a patient, for example, who took six meetings to understand why he had to check his glucose, capillary blood glucose, every day, you know? Just like I had a patient who understood everything by the second meeting, and she was so interesting that she summoned the courage to discuss it with the doctor that the doctor changed her entire treatment. And it’s what I told you, each patient takes time to realize and understand.”*

(F8)

The reports show that prior experience with PC and participation in CPD initiatives were fundamental to the implementation of the service. Most participants demonstrated an interest in clinical practice before the continuing education initiative, seeking out courses, materials, and experiences on their own. The training provided by the UFSJ project was valued as a space for technical qualification, sharing experiences, and strengthening professional performance. In contrast, participants suggested the need for greater integration between theory and clinical practice in the training.

*“If I hadn’t had this time, I might not have been so successful, because we need to study. I didn’t have the training to do this, nor the technical knowledge to do it. For a long time, I was deprived of this knowledge of medication. To perform any type of intervention, you have to understand the patient’s clinical condition a little better. So, I studied to be able to perform any type of intervention.”*

(F4)

### 3.2. Pharmaceutical Care in Professional Practice and Healthcare

In the interviewees’ perception, the **structuring of PC in PHC brought significant potential for the qualification** of health care and the appreciation of professional performance. Contributions mentioned included strengthening humanized and individualized patient care; strengthening PHC and **shared care; moving beyond the biomedical model and strengthening the pharmacist’s clinical role; and the production of health indicators** for municipalities.

- Qualified care and a patient-centered approach:

*“I believe that this clinical pharmacy work was created to focus on the patient, right? And also to improve the patient’s quality of life, both in prevention and treatment. Primary health care units are already overwhelmed with patient numbers, so we need to have this patient-centered approach… So I think this approach was missing, the presence of pharmacists was missing in primary care.”*

(F1)

- Strengthening PHC and shared care:

*“When patients connect with us, they significantly improve their treatment, the quality of their care. So, when other professionals see that we are doing this and the patient improves, it makes it easier for them to come in. And often, when we provide guidance here in the pharmacy, we don’t follow up with the patient, and then they return to make the same mistake. And when you do it within primary care, the staff see your knowledge, your capabilities, and they start wanting to even refer patients to you.”*

(F4)

- Moving beyond the biomedical model and strengthening the pharmacist’s clinical role:

*“Although we’re still in our infancy, we’ll continue… until we change the idea of centralizing everything around the doctor, things won’t move forward. Primary care is about caring, right? It’s about prevention. So, the pharmacist would be essential. It’s not a matter of choice, it’s a matter of being essential.”*

(F6)

- Production of health **indicators** and outcomes for municipalities

*“We’ve had very good responses, very good results. We had glycated hemoglobin levels go from 13.8% to 8%, 13.6% to 7.8%, so this shows that it was effective, it had good results, favorable results.”*

(F2)

Furthermore, the interviewees highlighted the impacts of the PC structure, such as concrete changes in both the pharmacist’s personal and professional satisfaction, in the credibility and recognition of the pharmacist’s work for the patient and for healthcare, and in patient satisfaction.

- Personal and professional satisfaction:

*“So, it was really good for both of us, you know? I changed as a professional, and she changed as a patient. I was important to her, she was important to me.”*

(F8)

- Recognition of the pharmacist by patients

*“Because patients used to associate the pharmacist with the medication itself. And today, that’s changed. They now associate the pharmacist with care. I think it’s even interesting. I overheard one day. I was leaving the clinic downtown and I heard two patients talking at the reception desk. There was a patient I had just seen, and this patient said to another: “But doesn’t your clinic have a pharmacist? Because here, the pharmacist takes care of us diabetics.” I thought her comment was so beautiful. So I think she stopped associating the pharmacist with the medicine, with the medication, and started associating the pharmacist with care.”*

(F7)

- Transformation of professional practice and continuity of the bond with the patient

*“There are people who have never been to the clinical pharmacy and say, ‘I wonder if she’ll give me medicine?’ And they immediately think that, right? And when they leave the pharmacy appointment there, they truly understand the importance of a clinical pharmacist and start asking for it […] So, when they come in and see me, wow, it’s nothing but praise. And being able to help, I’m so happy, right? Being able to help the patient.”*

(F3)

## 4. Discussion

The findings of this study reinforce that the implementation of PC in PHC is a dynamic process, strongly conditioned by the local context and the interactions between professionals, management, and users. The analysis of the interviews revealed that the consolidation of PC, like other clinical services, does not depend exclusively on specific training for pharmacists, but also on structural conditions, managerial support, and effective integration with the multidisciplinary team. This understanding aligns with the proposal of the **health education quadrilateral**, which emphasizes the inseparability of teaching, management, care, and social control in the consolidation of care practices [29].

The results indicate that the implementation of PC in the public health system is influenced by contextual and structural factors. Among these aspects, **interprofessional relationships** stand out, which are marked by varying degrees of integration, recognition, and cooperation in the daily work of teams. **The variety and complexity of these interactions** directly influence how pharmacists are included in care practices and how their clinical work is received by other professionals. This perspective is reinforced by other studies that indicate that the implementation of PC can be favored in settings with an integrated interdisciplinary team, especially when the pharmacist is previously included in the work process [19,30,31].

Furthermore, **resistance to change** on the part of the healthcare team is also reported in other studies and may be related to a lack of understanding of the pharmacist’s clinical role and their contributions to care [32,33,34]. This scenario highlights the need for attitudinal changes, continuous effort, and persistence—at the individual and organizational levels—as well as clear role definition and investment in communication and interprofessional integration strategies to promote transformations aligned with contemporary social demands [34,35].

Another point identified refers to **management support**, which, although mentioned as a facilitating element, was often limited to mere talk, without implying adequate material conditions. Studies found similar barriers regarding consultation time, availability of consultation area, and support staff [36,37,38]. Santos Júnior et al. (2018) emphasize that the lack of adequate physical infrastructure in healthcare facilities directly hinders privacy, patient bonding, and the quality of clinical care [38]. Insufficient human resources lead to an overload of administrative tasks and reduce the pharmacist’s focus on clinical activities [39]. This evidence reinforces the need for effective management actions to strengthen PC and expand its impact on healthcare services.

Strategies for establishing **agreements with patients** also proved challenging. The public’s lack of understanding of the pharmacist’s clinical role and the need for continued adherence to care required additional effort from professionals. Studies show that educational and supportive interventions can empower patients to self-manage their medications, identify medication-related challenges, and increase adherence, with a positive impact on treatment outcomes [16,36,40,41,42]. In this context, communication between health professionals and patients, combined with interdisciplinary approaches and educational strategies, constitutes a central element in overcoming resistance, promoting adherence and ensuring the safety and effectiveness of care [16,36,43].

Furthermore, prior training and **professional qualifications** are essential elements for consolidating the clinical role of pharmacists, especially in the context of the public health system, where **CPD** is considered a structuring strategy for transforming health practices [44,45]. According to Etukakpan et al. (2023), promoting continuous professional development, from graduation to throughout the career, can result in a qualified pharmaceutical workforce, which will assume its natural place in the health team [46].

In addition to training, reports reinforce that the implementation of PC has had significant impacts on professional practice and the **qualification of health care**. The **patient-centered** approach, highlighted by the participants, highlights the transition from a medication-focused model to person-centered care, reflecting the changes that the pharmacy profession is undergoing [47,48,49,50]. In this context, changes in healthcare processes, associated with the expansion of the biopsychosocial model and the redefinition of public policies, have driven this transition, interpreted in the literature as a “re-professionalization” of pharmacists in relation to patients [44,48,51]. This change does not imply an overlapping of roles, but rather the affirmation of a specific clinical role, in which the pharmacist becomes co-responsible for therapeutic outcomes and comprehensive care.

Another relevant aspect concerns the humanistic impacts related to **personal and professional satisfaction**, the **social recognition of pharmacists**, and the **transformation of daily practice**. These findings reinforce the idea that the implementation of PC not only improves care but also redefines professional identities and intersubjective relationships in PHC. In this sense, the literature indicates that pharmacists’ job satisfaction is strongly associated with good relationships with patients, professional autonomy, continuing education, and interaction with other members of the healthcare team, serving as a motivating factor for continued work [52,53].

Finally, although many of the factors identified are not new to the literature on the implementation of clinical services, the relevance of this study lies in demonstrating, through the voices of pharmacists themselves, that obstacles have persisted for decades, even in the face of successive training initiatives and incentives for clinical care. Since the 1990s, efforts have been made to consolidate clinical pharmaceutical practice. However, as Hepler and Strand (1990) emphasized, consolidating a clinical profession requires redefining roles and responsibilities, drawing inspiration from the practices and models of professions already working clinically [54]. Thus, by addressing these issues from the daily reality of professionals working in PHC, this study contributes to the recognition that CPD and other training initiatives, while essential, are not sufficient on their own to sustain the implementation of pharmaceutical care, which also depends on structural, organizational, and cultural change. These barriers should be recognized as persistent systemic challenges, rather than merely individual training gaps.

Regarding the study’s limitations, it is acknowledged that the findings reflect the specific reality of the context analyzed. Therefore, understanding the experiences of pharmacists who participated in the PC implementation process helps reveal nuances and lessons learned that can inspire other contexts. Although the number of participants should be interpreted in light of the qualitative design, it is important to acknowledge that only 8 of the 17 eligible pharmacists participated in the interviews. Nevertheless, those included had direct experience with the implementation process and provided sufficiently rich and diverse accounts to support the analysis of this specific context.

It is observed that, among the potential participants invited, those who did not respond to contact were precisely those who had not completed ImplanFarSUS, which suggests a potential selection bias and may have contributed to a greater representation of more engaged participants. However, it is important to note that the interviewed group also included pharmacists who had not completed the planned activities of the project, which partially mitigates the possibility of an exclusively positive portrayal of the process. It is also noteworthy that the data derive exclusively from the perspective of pharmacists, without considering the views of patients, managers, or other healthcare team professionals, which could broaden the understanding of the phenomenon. Because pharmaceutical care implementation is relational and organizational, its implications cannot be fully understood from pharmacists’ perspectives alone.

The potential, in contrast, is related to deepening issues related to the facilitating and hindering factors for PC implementation in a context subsequent to the PC implementation process. By analyzing the perceptions of pharmacists themselves, the study fills a gap in the literature on the implementation process and sheds light on the paths that must be taken to implement clinical services in the context of PC.

## 5. Conclusions

This study showed that implementing PC in PHC requires continuing education, institutional support, and integration with the multidisciplinary team. The findings demonstrate progress in the transition from a medication-centered model to person-centered care, resulting in greater PHC qualifications, strengthening the clinical role of pharmacists, and positive impacts on job satisfaction and social recognition. However, consolidating PC requires advancing CPD proposals, understanding it not only as a provision of specific training, but as an ongoing process of reflection and transformation of practices. Therefore, strengthening PC depends on consistent public policies and structural and pedagogical conditions that promote on-the-job learning and ensure the long-term sustainability and effectiveness of clinical services.

## Figures and Tables

**Table 1:** Sociodemographic and educational characteristics of the participants (n = 8).

Interviewee	Sex	Age Range (Years)	Type of University from Which Graduated	Post-Graduate	Time Since Graduation (Years)	Time Working in the Public Health System (Years)	Exclusive eMulti Pharmacist
F1	F	30–39	Public	Yes	6 to 10	0–5	Yes
F2	F	40–49	Public	No	More than 15	More than 15	No
F3	F	30–39	Private	Yes	11 to 15	0–5	No
F4	M	40–49	Public	Yes	More than 15	More than 15	No
F5	F	40–49	Public	Yes	More than 15	6–10	Yes
F6	F	40–49	Public	Yes	More than 15	11–15	No
F7	F	30–39	Private	Yes	6–10	6–10	Yes
F8	M	30–39	Private	Yes	11 to 15	11–15	No

Legend: eMulti: Multidisciplinary Teams in Primary Health Care.

## Data Availability

The datasets generated and/or analyzed during the current study are not publicly available due to ethical and confidentiality concerns. The full interview transcripts contain sensitive information that may compromise participant anonymity. All excerpts included in the manuscript were carefully selected and anonymized to ensure confidentiality. Access to limited data may be granted by the corresponding author upon reasonable request, subject to approval by the relevant ethics committee.
